# Supplementary material for: Differential privacy for medical deep learning: methods, tradeoffs, and deployment implications
Source: NPJ Digit Med. 2026 Jan 3;9:93. doi: 10.1038/s41746-025-02280-z (PMC12855931; doi:10.1038/s41746-025-02280-z)
Supplement: Supplementary file 1 — Supplementary Information [file 41746_2025_2280_MOESM1_ESM.pdf]

Differential privacy for medical deep learning: methods, tradeoffs, and deployment implications – Supplementary information

Marziyeh Mohammadi[1], Mohsen Vejdanihemmat[1], Mahshad Lotfinia[2], Mirabela Rusu[3,4], Daniel Truhn[2], Andreas Maier[1], Soroosh Tayebi Arasteh[1,2,3,4*]

[1]Pattern Recognition Lab, Friedrich-Alexander-Universität Erlangen-Nürnberg, Erlangen, Germany.
[2]Department of Diagnostic and Interventional Radiology, University Hospital RWTH Aachen, Aachen, Germany.
[3]Department of Radiology, Stanford University, Stanford, CA, USA.
[4]Department of Urology, Stanford University, Stanford, CA, USA.

*Corresponding author E-mail: soroosh.arasteh@rwth-aachen.de

Supplementary Tables and Figures

Section	Item	PRISMA-ScR checklist item	Status
**Title**			
Title	1	Identify the report as a scoping review.	✓
**Abstract**			
Structured summary	2	Provide a structured summary that includes (as applicable): background, objectives, eligibility criteria, sources of evidence, charting methods, results, and conclusions that relate to the review questions and objectives.	✓

Section	Item	PRISMA-ScR checklist item	Status
**Introduction**			
Rationale	3	Describe the rationale for the review in the context of what is already known. Explain why the review questions/objectives lend themselves to a scoping review approach.	✓
Objectives	4	Provide an explicit statement of the questions and objectives being addressed with reference to their key elements (e.g., population or participants, concepts, and context) or other relevant key elements used to conceptualize the review questions and/or objectives.	✓
**Methods**			
Protocol and registration	5	Indicate whether a review protocol exists; state if and where it can be accessed (e.g., a Web address); and if available, provide registration information, including the registration number.	✓
Eligibility criteria	6	Specify characteristics of the sources of evidence used as eligibility criteria (e.g., years considered, language, and publication status), and provide a rationale.	✓
Information sources	7	Describe all information sources in the search (e.g., databases with dates of coverage and contact with authors to identify additional sources), as well as the date the most recent search was executed.	✓
Search	8	Present the full electronic search strategy for at least 1 database, including any limits used, such that it could be repeated.	✓
Selection of sources of evidence	9	State the process for selecting sources of evidence (i.e., screening and eligibility) included in the scoping review.	✓

Section	Item	PRISMA-ScR checklist item	Status
Data charting process	10	Describe the methods of charting data from the included sources of evidence (e.g., calibrated forms or forms that have been tested by the team before their use, and whether data charting was done independently or in duplicate) and any processes for obtaining and confirming data from investigators.	✓
Data items	11	List and define all variables for which data were sought and any assumptions and simplifications made.	✓
Critical appraisal of individual sources of evidence	12	If done, provide a rationale for conducting a critical appraisal of included sources of evidence; describe the methods used and how this information was used in any data synthesis (if appropriate).	NA
Synthesis of results	13	Describe the methods of handling and summarizing the data that were charted.	✓
**Results**			
Selection of sources of evidence	14	Give numbers of sources of evidence screened, assessed for eligibility, and included in the review, with reasons for exclusions at each stage, ideally using a flow diagram.	✓
Characteristics of sources of evidence	15	For each source of evidence, present characteristics for which data were charted and provide the citations	✓
Critical appraisal within sources of evidence	16	If done, present data on critical appraisal of included sources of evidence (see item 12).	NA
Results of individual sources of evidence	17	For each included source of evidence, present the relevant data that were charted that relate to the review questions and objectives.	✓
Synthesis of results	18	Summarize and/or present the charting results as they relate to the review questions and objectives	✓

Section	Item	PRISMA-ScR checklist item	Status
**Discussion**			
Summary of evidence	19	Summarize the main results (including an overview of concepts, themes, and types of evidence available), link to the review questions and objectives, and consider the relevance to key groups.	✓
Limitations	20	Discuss the limitations of the scoping review process	✓
Conclusions	21	Provide a general interpretation of the results w.r.t the review questions and objectives, as well as potential implications and/or next steps.	✓
**Funding**			
Funding	19	Describe sources of funding for the included sources of evidence, as well as sources of funding for the scoping review. Describe the role of the funders of the scoping review.	✓

Supplementary Table 1: PRISMA-ScR checklist [1]

Database	Results	Search string
PubMed	63	(”differential privacy”[TIAB] OR ”differentially private”[TIAB] OR ”DP-SGD”[TIAB]) AND (”deep learning”[TIAB] OR ”machine learning”[TIAB] OR ”neural network”[TIAB]) AND (”medicine”[TIAB] OR ”medical”[TIAB] OR ”clinical”[TIAB] OR ”biomedical”[TIAB] OR ”health”[TIAB] OR ”EHR”[TIAB] OR ”imaging”[TIAB] OR ”radiology”[TIAB] OR ”text”[TIAB] OR ”speech”[TIAB]) NOT (review[PT])
	84	(”differential privacy” OR ”differentially private” OR ”DP-SGD”) AND (”deep learning” OR ”machine learning” OR ”neural network” OR ”artificial intelligence”) AND (”medical” OR ”medicine” OR ”clinical” OR ”biomedical” OR ”healthcare” OR ”EHR” OR ”imaging” OR ”radiology” OR ”MRI” OR ”CT” OR ”ultrasound” OR ”speech” OR ”text” OR ”video”) NOT (review[PT])
	417	(”differential privacy”[TIAB] OR ”differentially private”[TIAB] OR ”DP-SGD”[TIAB] OR ”privacy preserving”[TIAB] OR ”privacy-preserving”[TIAB] OR ”securing”[TIAB] OR ”safeguarding”[TIAB]) AND (”deep learning”[TIAB] OR ”machine learning”[TIAB] OR ”artificial intelligence”[TIAB] OR ”neural network”[TIAB]) AND (”medical”[TIAB] OR ”medicine”[TIAB] OR ”clinical”[TIAB] OR ”healthcare”[TIAB] OR ”biomedical”[TIAB] OR ”EHR”[TIAB] OR ”electronic health record”[TIAB] OR ”imaging”[TIAB] OR ”radiology”[TIAB] OR ”text”[TIAB] OR ”speech”[TIAB]) NOT (review[PT])
IEEE Xplore	676	(”differential privacy” OR ”differentially private” OR ”DP-SGD”) AND (”deep learning” OR ”machine learning” OR ”neural network”) AND (”medicine” OR ”medical” OR ”clinical” OR ”healthcare” OR ”biomedical” OR ”EHR” OR ”imaging” OR ”radiology” OR ”text” OR ”speech”)
Web of Science	494	”differential privacy” AND (”deep learning” OR ”machine learning”) AND (medical OR medicine OR clinical OR healthcare OR biomedical OR imaging OR EHR OR radiology OR text OR speech)
	571	(”differential privacy” OR ”differentially private” OR ”DP-SGD”) AND (”deep learning” OR ”machine learning” OR ”artificial intelligence” OR ”neural network”) AND (medical OR medicine OR clinical OR healthcare OR biomedical OR imaging OR EHR OR radiology OR text OR speech)
ACM Digital Library	999	”differential privacy” AND (”deep learning”) AND (medical OR EHR OR imaging)
	938	”differential privacy” AND (”artificial intelligence”) AND (medical OR clinical OR healthcare OR EHR OR imaging OR radiology OR biomedical)

Supplementary Table 2: Search results from different databases

Supplementary References

[1] Tricco, A.C., Lillie, E., Zarin, W., O'Brien, K.K., Colquhoun, H., Levac, D., Moher, D., Peters, M.D., Horsley, T., Weeks, L., *et al.*: Prisma extension for scoping reviews (prisma-scr): checklist and explanation. Annals of internal medicine **169**(7), 467–473 (2018)
